# Solvent-Free Copper-Catalyzed Azide-Alkyne Cycloaddition under Mechanochemical Activation

**DOI:** 10.3390/molecules20022837

**Published:** 2015-02-09

**Authors:** Laura Rinaldi, Katia Martina, Francesca Baricco, Laura Rotolo, Giancarlo Cravotto

**Affiliations:** 1 Dipartimento di Scienza e Tecnologia del Farmaco and NIS—Centre for Nanostructured Interfaces and Surfaces, University of Turin, Via P. Giuria 9, Turin 10125, Italy; E-Mails: laura.rinaldi@unito.it (L.R.); katia.martina@unito.it (K.M.); francesca.baricco@hotmail.it (F.B.); laura.rotolo@unito.it (L.R.); 2 Dipartimento di Biotecnologie Molecolari e Scienze per la Salute, University of Turin, Via Quarello 15, Turin 10125, Italy

**Keywords:** click reaction, copper catalyzed alkyne-azide cycloaddition, ball mill, solvent-free reaction, mechanochemistry

## Abstract

The ball-mill-based mechanochemical activation of metallic copper powder facilitates solvent-free alkyne-azide click reactions (CuAAC). All parameters that affect reaction rate (*i.e*., milling time, revolutions/min, size and milling ball number) have been optimized. This new, efficient, facile and eco-friendly procedure has been tested on a number of different substrates and in all cases afforded the corresponding 1,4-disubstituted 1,2,3-triazole derivatives in high yields and purities. The final compounds were isolated in almost quantitative overall yields after simple filtration, making this procedure facile and rapid. The optimized CuAAC protocol was efficiently applied even with bulky functionalized β-cyclodextrins (β-CD) and scaled-up to 10 g of isolated product.

## 1. Introduction

In the 1960s, Huisgen and co-workers extensively studied the 1,3-dipolar cycloaddition reaction of azides and alkynes [1,2]. In Denmark, Meldal *et al.* [3] reported the Cu(I)-catalyzed alkyne-azide click reaction (CuAAC) at the same time as Fokin and Sharpless [4] in the U.S. The use of copper salts as the catalyst exclusively afforded 1,4-disubstituted 1,2,3-triazoles, whereas the uncatalyzed reaction provided mixtures of 1,4- and 1,5-triazole regioisomers, while also necessitating much higher temperatures. The CuAAC is considered to be the “click chemistry” reaction *par excellence*. In 2001, Sharpless coined this term to describe a set of powerful bond-forming reactions with almost complete orthogonality (*i.e*., which do not interfere with other chemical functionalities) [5]. CuAAC has been used in many fields, has become a true interdisciplinary reaction and can boast of extremely wide applicability [6,7,8,9]. The required Cu(I) catalyst is usually generated *in situ* via the reduction of a Cu(II) salt with sodium ascorbate.

Enabling techniques such as microwave heating (MW), ultrasound irradiation (US) and mechanochemical activation are the most reliable energy sources with which to activate catalysts and promote chemical reactions [10,11,12,13]. The study of highly efficient MW- and US-promoted CuAAC protocols has become an important goal [14] and a wide range of compounds has been synthesized under non-conventional conditions [15], while increasing interest in biological applications has lead to MW being used to obtain multimeric peptides and peptidomimetcs [16,17], to modify nucleotides or nucleic acid [18,19] and to synthesize dendrimeric multicarriers [20,21]. MW-assisted CuAAC have been applied to material grafting and polymer functionalization [22,23,24,25], as well as for the preparation of functional polytriazoles [26].

Sonochemical reactions fulfil green chemistry requirements and thus cause a sizeable reduction in energy consumption [27]. In particular, US has found its main domain in heterogeneous catalysis and in metal surface activation [28,29,30]. Metallic copper is one of the cheapest solid catalysts that in a relatively longer reaction time can afford very clean final products after simple removal of copper powder or turnings [31,32,33]. Our experience has shown us that US irradiation smoothly activates the redox process between metallic Cu and Cu_2_O on the metal surface, generating Cu(I) species. This means that simple copper turnings can be used as the catalyst of choice in Huisgen 1,3-dipolar cycloadditions [34,35]. Despite the different reaction environment, a combination of US and mechanochemical grinding can convert mechanical energy into a chemical outcome [36], and so planetary mills (PM) can be exploited to activate the metallic copper surface. The amount of energy which can be imparted to a system under mechanical activation can be sufficient to break chemical bonds [37]. Although the full extent of the technique applicability still remain uncovered [38,39] the use of PM for metal activation is well known [40], with a number of mechanochemical organic reactions [41], among them oxidations [42], Knovenagel and domino condensation reactions [43], metal-catalyzed cycloadditions [44,45,46]. PM has been exploited as a tool to reduce the energy demand in C-C couplings such as Suzuki, Heck, Sonogashira reactions [47] and possibly at least in part replace palladium catalysts with less expensive transition metals. Few examples of mechanochemical copper-catalysed organic reactions have been reported by Thorwirth *et al.* (CuAAC with a classic Cu(OAc)_2_/ascorbate system) [48] and by Friščić *et al*., (Cu(I)-catalysed C-N couplings) [49]. On the basis of our experience in sonochemistry, Cu(0) powder could also be a good source of Cu(I) catalyst for CuAAC under mechanochemical conditions [30,50].

Green chemistry utilizes a set of principles that favours the reduction or elimination of organic solvents [51], prefers heterogeneous catalysis, minimizes waste production and energy consumption. The advantages of non-conventional techniques such as MW, US and mechanochemistry either in benign reaction media, such as water, or in solventless conditions have been well documented in the literature [52,53]. In this work, we aimed to design a scalable mechanochemical CuAAC protocol using metallic copper powder under solvent-free conditions. The study included the optimization of all parameters that influence reaction rate and product yields.

## 2. Results and Discussion

**Scheme 1:**
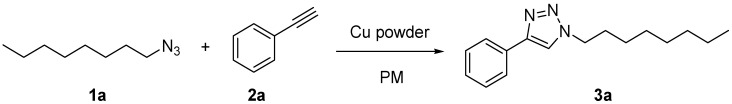
CuAAC of octyl azide (**1a**) with phenylacetylene (**2a**) forming 1-octyl-4-phenyl-*1H*-1,2,3-triazole (**3a**).

As depicted in Scheme 1, the CuAAC with octyl azide (**1a**) and phenylacetylene (**2a**) has been selected as our model reaction. It was chosen because of its resistance to mechanical shock. The reaction was first studied under conventional conditions: the reagents were dissolved in *t-*BuOH:H_2_O 1:1 and the reaction was stirred at 70 °C for 20 h (full conversion by GC-MS analysis).

**Figure 1:**
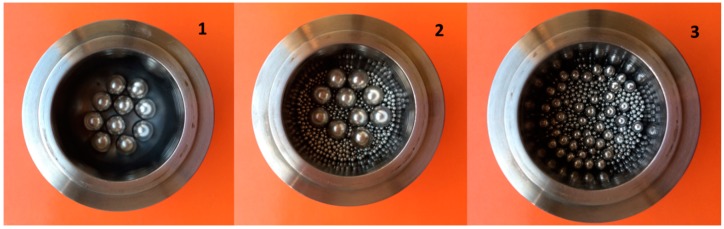
Jar and balls (Entries 1–3 Table 1).

The first attempt to mechanochemicalCuAAC was carried out in a stainless steel jar (50 mL) with 10 balls (10 mm Ø), octyl azide (1 mmol) and phenylacetylene (1 mmol) Cu powder (1 mmol), and SiO_2_ (5 g) used as grinding auxiliary. After 30 min at 650 rpm the triazole derivative was obtained in a 67% yield. As already reported in the literature, stress energy can be considered an energy distribution which sums all single stress events [38]. The number of stress events and the stress frequency, which are correlated to ball diameter and the number of balls, influence reaction outcome. Ball dimension and number were optimized while other parameters were fixed (time = 30 min, rotation speed = 650 rpm, auxiliary = 5 g of SiO_2_) (See Table 1 and Figure 1).

**Table 1:** Screening of ball features.

Entry	Ball Number			Active Surface Area ^b^ (mm^2^)	Yield (%) ^a^
	Small (Ø = 2 mm)	Medium (Ø = 5 mm)	Big (Ø = 10 mm)		
1	none	none	10	10,666	67
2	625	none	10	18,520	80
3	1500	48	none	30,144	99

^a^: Isolated yield; ^b^: Active surface area = surface_balls_ + surface_jar_.

Three different reaction conditions were compared. Small balls were added to the large 10 mm balls to fill empty space and increase the active surface area (Entry 2, Table 1). As shown in Table 1, the increase in effective energy transfer to the mill boosted the **3a** yield from 67% to 80%. A further improvement was achieved in a third experiment: the overall mass of the milling balls was kept constant and ball size and ball number was varied. The yield reached 99% as a result of the increase in grinding material active surface area.

**Figure 2:**
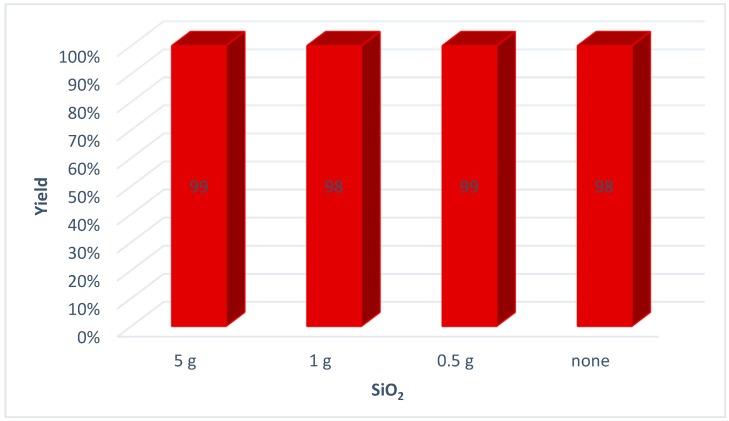
Tests with different grinding auxiliary amounts. *Reaction conditions*: Octyl azide (**1a**) (1 mmol), phenylacetylene (**2a**) (1 mmol), Cu powder (1 mmol), SiO_2_; stainless steel jar (50 mL), 1500 small balls (Ø = 2 mm) and 48 medium balls (Ø = 5 mm).

The grinding of liquids in PM can be performed in two ways: either by pre-freezing the reaction mixture below the eutectic melting point or by using grinding auxiliaries. The first technique is disadvantageous for economic reasons and because the compounds are rapidly heated during the milling process. The various amounts of silica (5 g, 1 g, 0.5 g) that were added to the reaction mixture did not affect the reaction outcome. The same yield was obtained in the absence of a grinding auxiliary (Figure 2).

**Figure 3:**
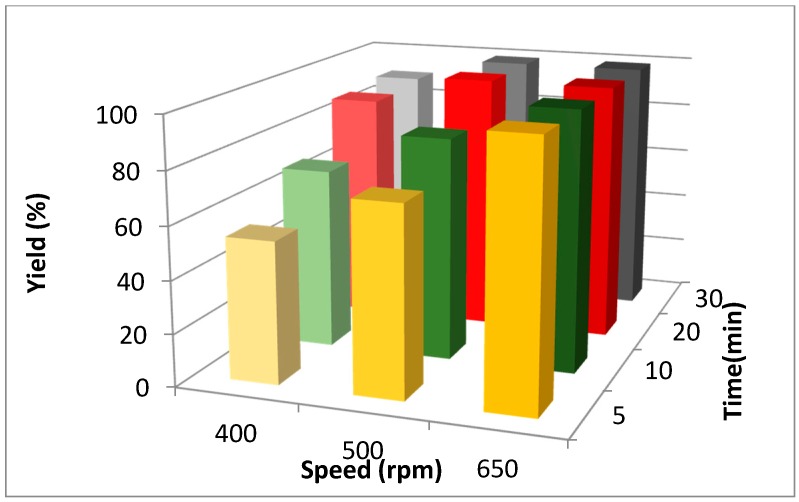
Influence of rotation frequency and milling time on product yield (**3a**). *Reaction conditions*: Octyl azide (**1a**) (1 mmol), phenylacetylene (**2a**) (1 mmol), Cu powder (1 mmol); stainless steel jar (50 mL), 1500 small balls (Ø = 2 mm) and 48 medium balls (Ø = 5 mm).

Two other relevant parameters were investigated, namely reaction time and PM rotation frequency (rpm, min^−1^). An increase in kinetic energy promoted the cycloaddition and a higher rotational speed enhanced conversion (Figure 3). Complete conversion was achieved in only 5 min at 650 rpm.

Using the optimized procedure (1 eq. Cu powder, 650 rpm, 5 min) as a basis, the octyl azide and phenylacetylene reaction was scaled-up to 10 mmol in a 50 mL jar. As depicted in Table 2, a 95% reaction yield was observed and mechanical energy transfer efficacy was confirmed in the planetary ball mill, even in the presence of small amounts of Cu powder (630 mg *vs.* 2.88 mL of reagents). In addition, the reaction was repeated on a larger scale (50 mmol) using a 250 mL jar charged with 10 balls (30 mm Ø) and 48 medium balls (5 mm Ø), in order to broaden the scope of the study and evaluate the versatility of the protocol. The isolated yield was around 95% and gave *ca.* 10 g of 1-octyl-4-phenyl-*1H*-1,2,3-triazole.

**Table 2:** Scale-up of mechanochemical CuAAC.

Entry	Octyl Azide (1a)		Yield ^a^	
	mmol	g	%	g
1 ^b^	1	0.1552	99	0.254
2 ^b^	10	1.5524	98	2.519
3 ^c^	50	7.7620	95	12.208

^a^: Isolated yield; ^b^: 1 eq Cu powder, 5 min, 650 rpm; 50 mL jar, 1500 small balls (Ø = 2 mm) and 48 medium balls (Ø = 5 mm); ^c^: 1 eq Cu powder, 5 min, 650 rpm; 250 mL jar, 48 medium balls (Ø = 5 mm) and 10 very large balls (Ø = 30 mm).

In an attempt to further confirm the versatility of the method, a selection of different benzyl azides and several alkynes were tested and the stability of iodo- and chloro- substituted phenyl moieties were thus confirmed (Entries 2 and 3, Table 3). As an efficient mechanochemical Knoevenagel-condensation has already been reported [52], alkynylalcohol was tested in order to evaluate the hydroxyl group’s susceptibility to dehydratation towards conjugated π double bonds (please check meaning) (Entry 9, Table 3). A diyne was tested so as to explore the efficiency of the protocol in the preparation of dimers.

As depicted in Table 3, all the reactions afforded pure triazole derivatives in high yields via simple Cu powder filtration. The high efficiency of the method was proven by the fast double 1,3-dipolar cycloadditions (5 min) of entry 10 (Table 3).

**Table 3:** Synthetic results of mechanochemical CuAAC ^a^.

Entry	Alkyne	Azide	Product	Yield ^b^ % (Conv. ^c^)
1 ^d^	**2a**	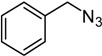 **1b**	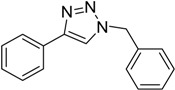 **3b**	98 (>99)
2 ^d^	**2a**	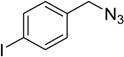 **1c**	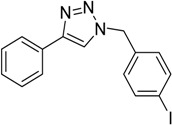 **3c**	97 (>99)
3	**2a**	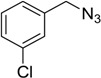 **1d**	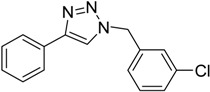 **3d**	95 (>99)
4 ^d^	**2a**	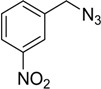 **1e**	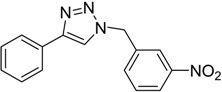 **3e**	91 (95)
4 ^d^	**2a**	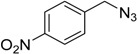 **1f**	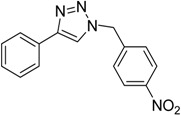 **3f**	94 (97)
5 ^d^	**2a**	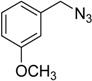 **1g**	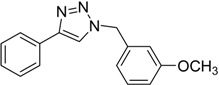 **3g**	97 (>99)
6	**2a**	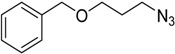 **1h**	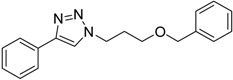 **3h**	90 (92)
7 ^d^	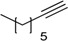 **2b**	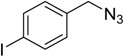 **1i**	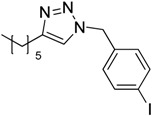 **3i**	98 (>99)
8	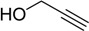 **2c**	**1a**	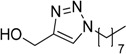 **3j**	92 (96)
9	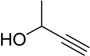 **2d**	**1a**	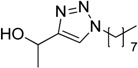 **3k**	88 (91)
10 ^e^	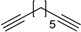 **2e**	**1a**	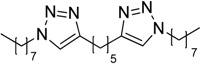 **3l**	98 (>99)
11 ^f^	**2a**	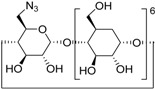 **1j**	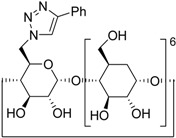 **3m**	81

^a^: *Reaction conditions*: azide **1** (1 mmol), alkyne **2** (1 mmol), Cu powder (1 mmol), 5 min, 650 rpm; stainless steel jar (50 mL), 1500 small balls and 48 medium balls; ^b^: Isolated yield, compound purity proved by ^1^H-NMR and ^13^C-NMR (see Supporting Info); ^c^: Determined by GC-MS; ^d^: Reaction time 10 min; ^e^: Excess **1a** (2 mmol); ^f^: *Reaction condition*: 0.1 mmol **1j** (6-monoazido-β-CD) (0.1 mmol), **2a** (0.5 mmol), Cu powder (0.1 mmol), 30 min.

CuAAC has been widely used in cyclodextrin (CD) functionalization, however Cu(II) salts tend to generate green-greyish coloured CD adducts because of the CD cavity’s excellent cation complexation properties [53]. This means that time-consuming competitive chelant purification methods become necessary. This drawback can be avoided by means of a solid supported Cu catalyst or using metallic copper. We have seen [35,54], that the click reaction afforded the desired 6-monotriazolyl-β-CD derivative when reacted in DMF in the presence of copper power under US or a combined MW/US irradiation. Gratifyingly, even better results can be achieved in PM (30 min) in solvent-free conditions, giving the CD derivatives as white powders and in high yields, as shown in Table 3 (entry 11). Considering the typical complications that occur in CD functionalization (low yield and low regioselectivity), the outstanding efficiency of CuAAC under mechanochemical activation may open the door to a totally new synthetic approach.

## 3. Experimental Section

### 3.1. General Information

All chemicals were purchased from Sigma-Aldrich (Milan, Italy) and used without further purification. Cu reduced powder RPE, purity >98%, was purchased from Carlo Erba (Milan, Italy). β-CD was kindly provided by Wacker Chemie (München, Germany). Reactions were monitored by TLC on Merck 60 F254 (0.25 mm) plates (Milan, Italy), which were visualized by UV inspection and/or by heating after a spraying with 5% H_2_SO_4_ in ethanol or phosphomolybdic acid. Mechanochemical reactions were carried out in a Planetary Ball Mill (PM100 Retsch GmbH, Haan, Germany) using either 50 or 250 mL grinding jars and milling balls (both made in stainless steel). NMR spectra (300 MHz and 75 MHz for ^1^H and ^13^C, respectively) were recorded on a Bruker 300 Avance instrument (Milan, Italy) at 25 °C. Chemical shifts were calibrated to the residual proton and carbon resonances of the solvent; DMSO-*d*_6_ (δH = 2.54, δC = 39.5), CDCl_3_ (δH = 7.26, δC = 77.16). Chemical shifts (δ) are given in ppm, and coupling constants (*J*) in Hz. GC-MS analyses were performed in a GC Agilent 6890 (Agilent Technologies, Santa Clara, CA, USA) that was fitted with a mass detector Agilent Network 5973, using a 30 m capillary column, i.d. of 0.25 mm and film thickness 0.25 μm. GC conditions were: injection split 1:20, injector temperature 250 °C, detector temperature 280 °C. Gas carrier: helium (1.2 mL/min), temperature program: from 70 °C (2 min) to 300 °C at 5 °C/min. HRMS was determined using a MALDI-TOF mass spectra (Bruker Ultraflex TOF mass spectrometer, Milan, Italy).

### 3.2. General Reaction Protocols

#### 3.2.1. General Procedure A for Alkyne-Azide Click Reaction (Preparation of **3a**, **3d**, **3h**)

The milling jar (50 mL; stainless steel) were equipped with 1500 milling balls (d = 2 mm, stainless steel) and 48 medium balls (d = 5 mm, stainless steel). Afterwards the alkyne (1 mmol), the azide (1 mmol) and Cu powder (1 mmol, 63 mg) were added in the given order. Milling was accomplished at 650 rpm for 5 min. After cooling of the milling jar to room temperature, the crude products were filtered on Büchner funnel with a sintered glass disc using diethyl acetate (3 × 10 mL). The solvent was evaporated in vacuum, the crude products were dried and analyzed by GC-MS, ^1^H-, ^13^C-NMR spectroscopy and MALDI-TOF mass spectrometry after dissolution in an appropriate solvent.

#### 3.2.2. General Procedure B for Alkyne-Azide Click Reaction (Preparation of **3b**, **3c**, **3e**, **3f**, **3g**, **3i**)

The milling jar (50 mL; stainless steel) were equipped with 1,500 milling balls (d = 2 mm, stainless steel) and 48 medium balls (d = 5 mm, stainless steel). Afterwards the alkyne (1 mmol), the azide (1 mmol) and Cu powder (1 mmol, 63 mg) were added in the given order. Milling was accomplished at 650 rpm for 10 min. After cooling of the milling jar to room temperature, the crude products were filtered on Büchner funnel with a sintered glass disc using diethyl acetate (3 × 10 mL). The solvent was evaporated in vacuum, the crude products were dried and analyzed by GC-MS, ^1^H-, ^13^C-NMR spectroscopy and MALDI-TOF mass spectrometry after dissolution in an appropriate solvent.

#### 3.2.3. General Procedure C for Alkyne-Azide Click Reaction (Preparation of **3l**)

The milling jar (50 mL; stainless steel) were equipped with 1,500 milling balls (d = 2 mm, stainless steel) and 48 medium balls (d = 5 mm, stainless steel). Afterwards the alkyne (1 mmol), the azide (2 mmol) and Cu powder (1 mmol, 63 mg) were added in the given order. Milling was accomplished at 650 rpm for 5 min. After cooling of the milling jar to room temperature, the crude product was filtered on Büchner funnel with a sintered glass disc using diethyl acetate (3 × 10 mL). The solvent was evaporated in vacuum, the crude products were dried and analyzed by GC-MS, ^1^H-, ^13^C-NMR spectroscopy and MALDI-TOF mass spectrometry after dissolution in an appropriate solvent.

#### 3.2.4. General Procedure D for Alkyne-Azide Click Reaction (Preparation of **3m**)

The milling jar (50 mL; stainless steel) were equipped with 1500 milling balls (d = 2 mm, stainless steel) and 48 medium balls (d = 5 mm, stainless steel). Afterwards the alkyne (0.5 mmol), the azide (0.1 mmol) and Cu powder (0.1 mmol, 6.3 mg) were added in the given order. Milling was accomplished at 650 rpm for 30 min. After cooling of the milling jar to room temperature, the crude product was was purified by flash chromatography on reverse phase (RP18, water-methanol). The solvent was evaporated in vacuum, the crude products were dried and analyzed by GC-MS, ^1^H, ^13^C-NMR spectroscopy and MALDI-TOF mass spectrometry after dissolution in an appropriate solvent.

## 4. Conclusions

In conclusion, we have developed an efficient and versatile mechanochemical CuAAC procedure using copper powder as a catalyst. The reaction was performed in a planetary ball mill without solvent and we have proven that the method is fast and efficient, affording the products in high yield after a simple work-up. The scaling-up of the octyl azide and phenylacetylene reaction followed a simple numbering-up procedure from milligrams to multigram scale production, and afforded the corresponding triazole derivative in an almost quantitative yield and a short reaction time (5 min).

## Supplementary Materials

Supplementary materials can be accessed at: http://www.mdpi.com/1420-3049/20/02/2837/s1.
