# Supplementary material for: Solvent-Free Copper-Catalyzed Azide-Alkyne Cycloaddition under Mechanochemical Activation
Source: Molecules. 2015 Feb 9;20(2):2837–49. doi: 10.3390/molecules20022837 (PMC6272186; doi:10.3390/molecules20022837)
Supplement: Supplementary file 1 [file molecules-20-02837-s001.pdf]

Supplementary Information

Analytical Data of the Products

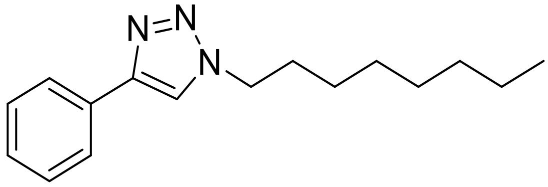

1-Octyl-4-phenyl-1H-1,2,3-triazole (**3a**) [1]: ^{1}H-NMR (300 MHz, $CDCl_3$): δ 7.75 (2 H, d, J = 9 Hz), 7.70 (1 H, s), 7.38–7.24 (3 H, m), 4.33 (2 H, t, J = 7.5 Hz), 1.90–1.85 (2 H, m), 1.35–1.12 (10 H, m), 0.80 (3 H, t, J = 6 Hz) ppm; ^{13}C-NMR (75 MHz, $CDCl_3$): δ 147.8, 130.9, 128.9, 128.2, 125.8, 119.5, 50.6, 31.8, 30.5, 29.2, 29.1, 26.6, 22.7, 14.2 ppm. *m/z* (MALDI-TOF MS): calcd for $C_{16}H_{23}N_3$ $[M+H]^+$: 258.1891, found: 258.1893.

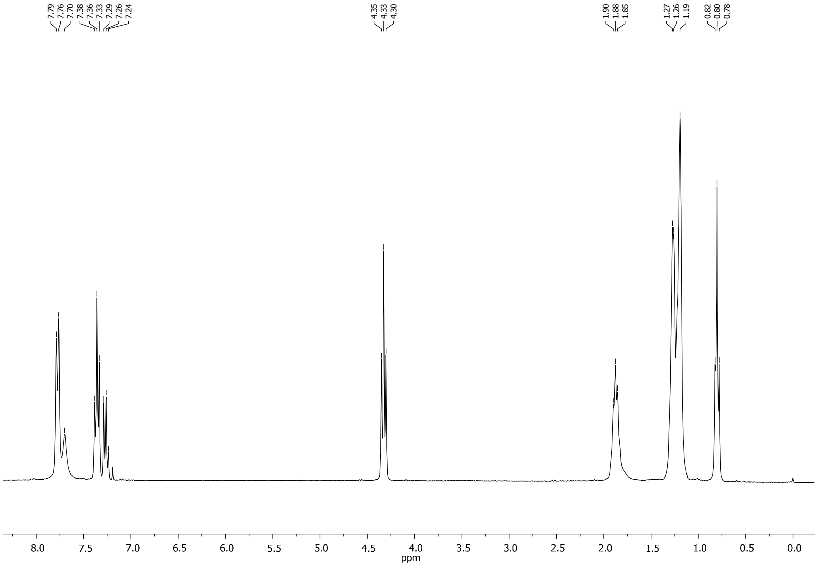

Figure S1. ^{1}H NMR (300 MHz, $CDCl_3$) product **3a**.

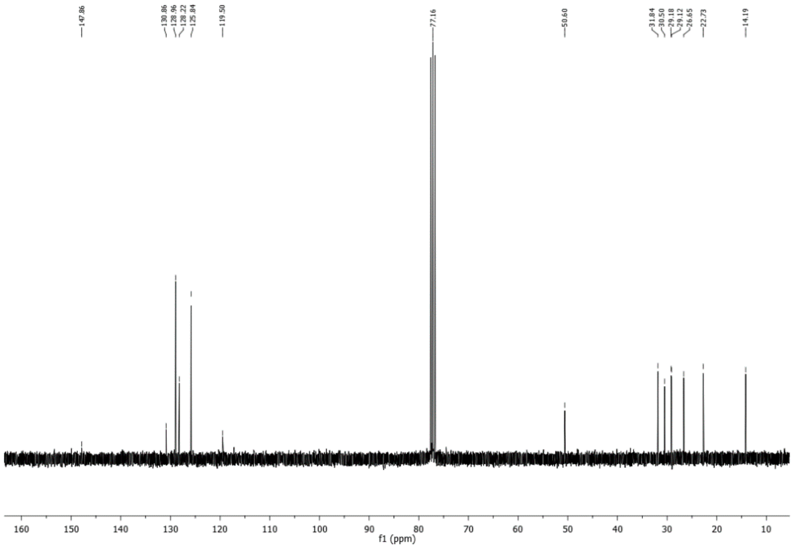

Figure S2. ^{13}C-NMR (75 MHz, $CDCl_3$) product **3a**.

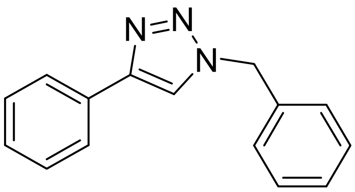

1-Benzyl-4-phenyl-1H-1,2,3-triazole (**3b**) [2]: ^{1}H-NMR (300 MHz, CDCl$_3$): δ 7.72 (2 H, d, *J* = 6 Hz), 7.59 (1 H, s), 7.34–7.21 (8 H, m), 5.49 (2 H, s) ppm; ^{13}C-NMR (75 MHz, CDCl$_3$): δ 148.6, 135.1, 130.9, 129.6, 129.2, 129.2, 128.6, 128.5, 126.1, 120.0, 54.7 ppm. *m/z* (MALDI-TOF MS): calcd for $C_{15}H_{13}N_3$ [M+H]$^+$: 236.1109, found: 236.1105.

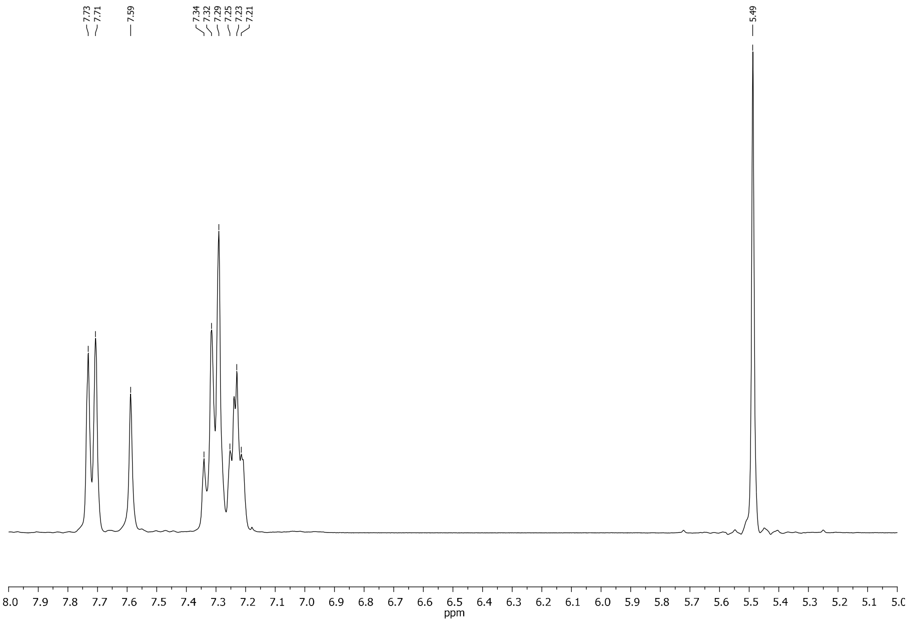

Figure S3. ^{1}H-NMR (300 MHz, CDCl$_3$) product **3b**.

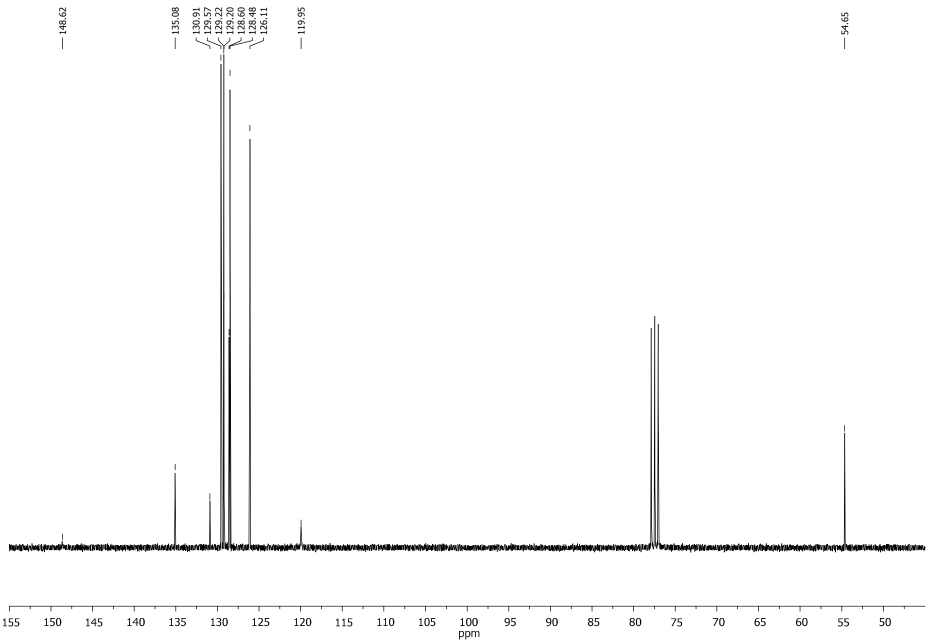

Figure S4. ^{13}C-NMR (75 MHz, CDCl$_3$) product **3b**.

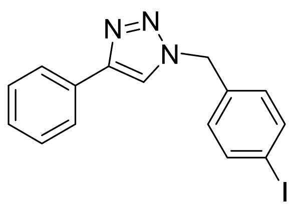

1-(4-Iodobenzyl)-4-phenyl-1H-1,2,3-triazole (**3c**) [3]: ^{1}H-NMR (300 MHz, $CDCl_3$): δ 7.79 (2 H, d, *J* = 4.5 Hz), 7.76–7.63 (3 H, m), 7.4–7.29 (3 H, m), 7.04 (2 H, d, *J* = 6 Hz), 5.51 (2 H, s) ppm; ^{13}C-NMR (75 MHz, $CDCl_3$): δ 150.1, 138.7, 134.8, 130.8, 130.2, 129.3, 128.7, 126.1, 119.9, 95.0, 54.0 ppm. *m/z* (MALDI-TOF MS): calcd for $C_{15}H_{12}IN_3$ $[M+H]^+$: 362.0075, found: 362.0074.

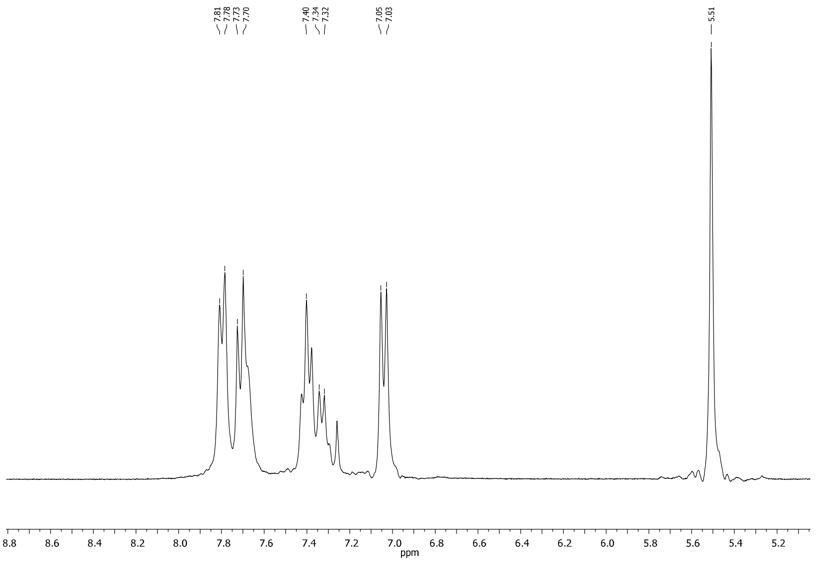

Figure S5. ^{1}H-NMR (300 MHz, $CDCl_3$) product **3c**.

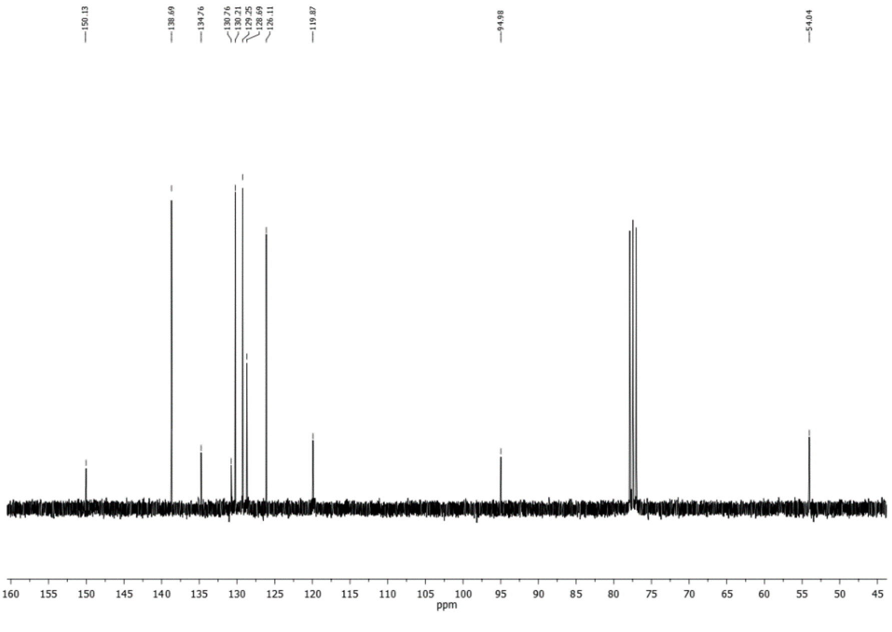

Figure S6. ^{13}C-NMR (75 MHz, $CDCl_3$) product **3c**.

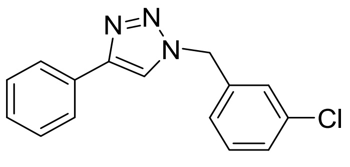

1-(3-Chlorobenzyl)-4-phenyl-1H-1,2,3-triazole (**3d**) [4]: ^{1}H-NMR (300 MHz, $CDCl_3$): δ 7.73 (2 H, d, *J* = 9 Hz), 7.63 (1 H, s), 7.41–7.10 (7 H, m), 5.47 (2 H, s) ppm; ^{13}C-NMR (75 MHz, $CDCl_3$): δ 148.8, 137.1, 135.4, 130.9, 130.8, 129.4, 129.3, 128.7, 128.5, 126,5, 126,1, 120.0, 53.9 ppm. *m/z* (MALDI-TOF MS): calcd for $C_{15}H_{12}ClN_3$ $[M+H]^+$: 270.0719, found: 270.0724.

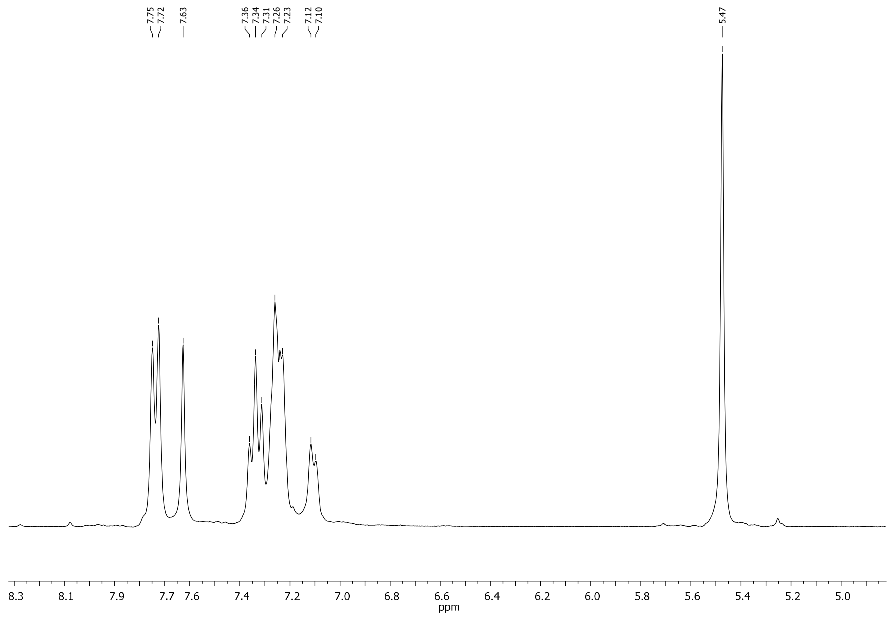

Figure S7. ^{1}H-NMR (300 MHz, $CDCl_3$) product **3d**.

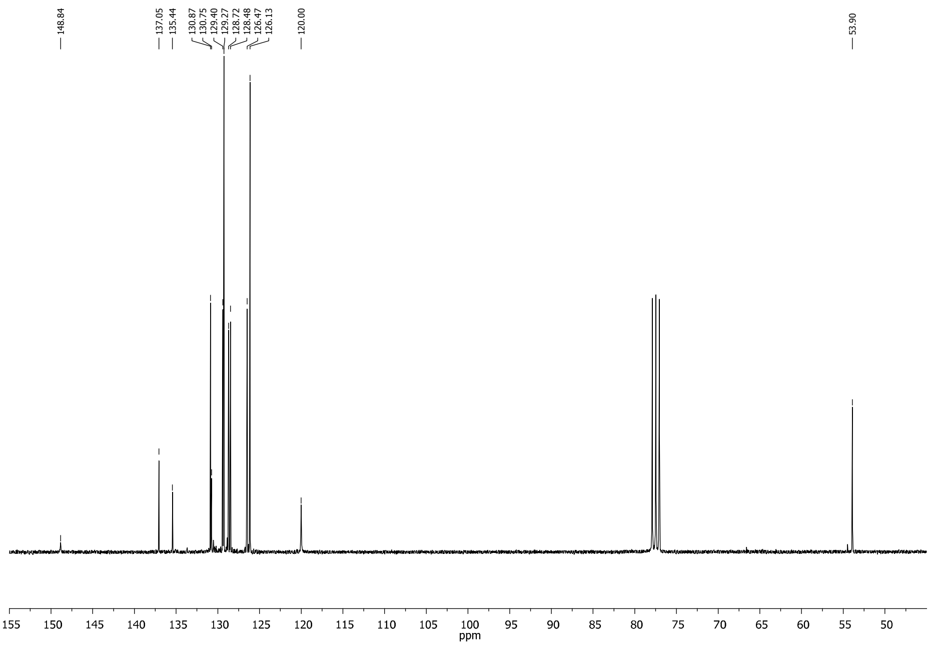

Figure S8. ^{13}C-NMR (75 MHz, $CDCl_3$) product **3d**.

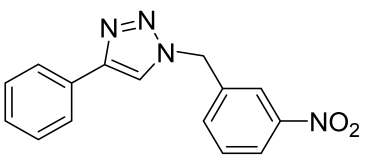

1-(3-Nitrobenzyl)-4-phenyl-1H-1,2,3-triazole (**3e**) [5]: ^{1}H-NMR (300 MHz, $CDCl_3$): δ 8.19 (2 H, b s), 7.79 (2 H, d, J = 3 Hz), 7.64–7.53 (3 H, s), 7.42–7.31 (3 H, m), 5.68 (2 H, s) ppm; ^{13}C-NMR (75 MHz, $CDCl_3$): δ 149.0, 148.9, 137.2, 134.3, 130.8, 130.5, 129.3, 128.9, 126.2, 124.2, 123.3, 120.2, 53.6 ppm. *m/z* (MALDI-TOF MS): calcd for $C_{15}H_{12}N_4O_2$ $[M+H]^+$: 281.0960, found: 281.0964.

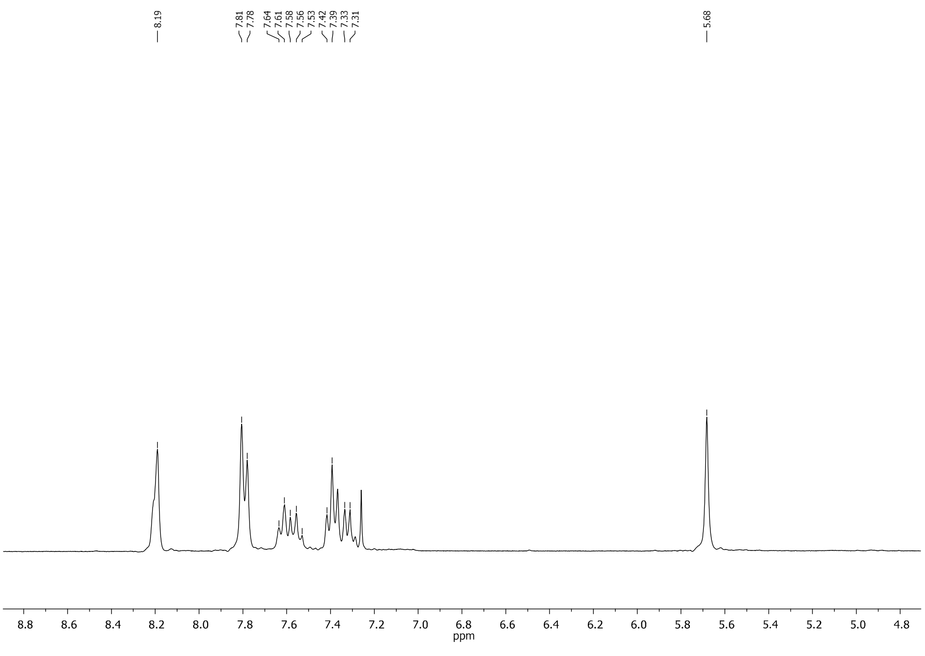

Figure S9. ^{1}H-NMR (300 MHz, $CDCl_3$) product **3e**.

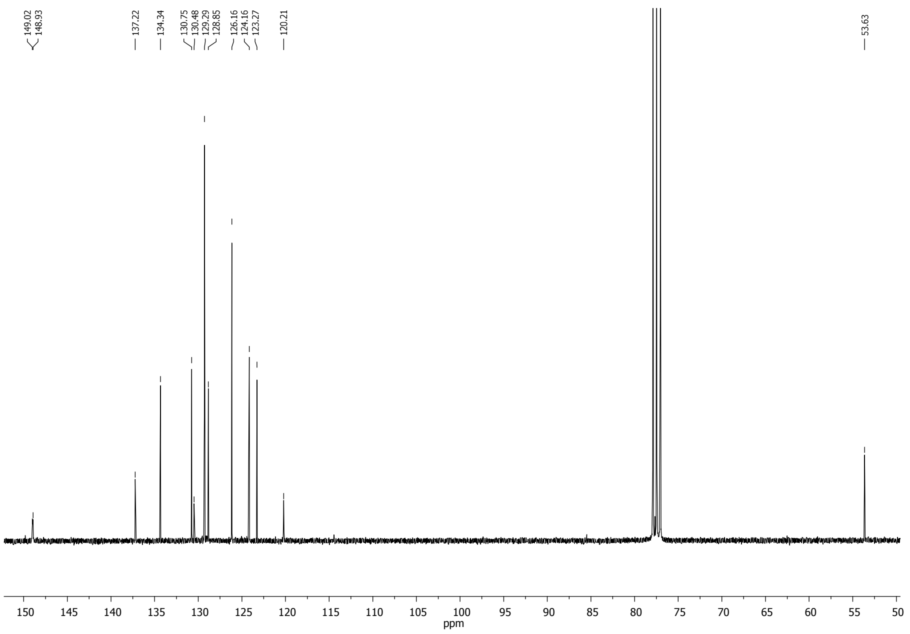

Figure S10. ^{13}C-NMR (75 MHz, $CDCl_3$) product **3e**.

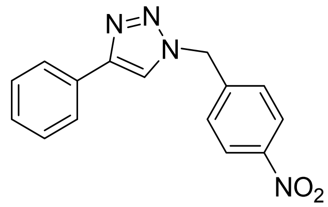

1-(4-Nitrobenzyl)-4-phenyl-1H-1,2,3-triazole (**3f**) [5]: ^{1}H-NMR (300 MHz, DMSO-d_6): δ 8.71 (1 H, s), 8.26 (2 H, d, J = 4.5 Hz), 7.87 (2 H, d, J = 3 Hz), 7.58 (2 H, d, J = 3 Hz), 7.46 (2 H, t, J = 9 Hz) 7.35 (1 H, t, J = 6 Hz) 5.85 (2 H, s) ppm; ^{13}C-NMR (75 MHz, DMSO-d_6): δ 147.2, 146.7, 143.4, 130.4, 129.3, 129.9, 128,8, 125.2, 123.9, 121.9, 52.1 ppm. m/z (MALDI-TOF MS): calcd for $C_{15}H_{12}N_4O_2$ [M+H]$^+$: 281.0960, found: 281.0958.

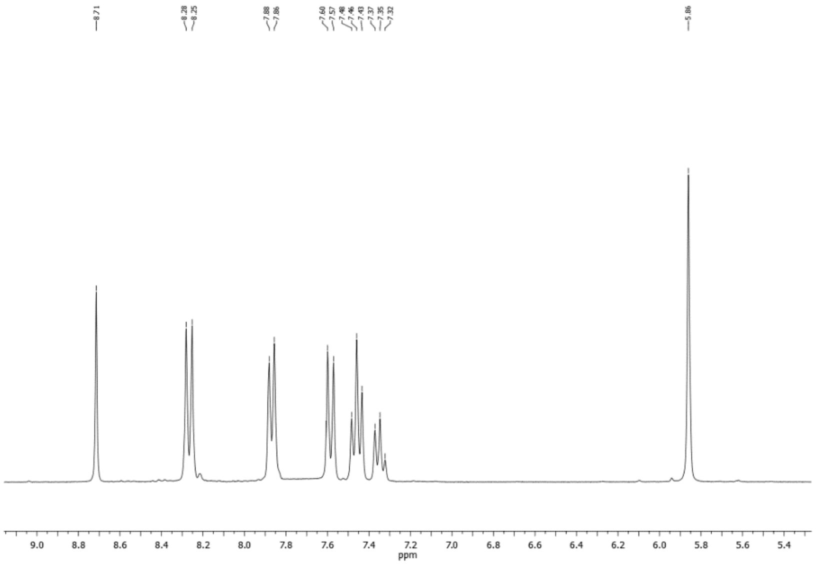

Figure S11. ^{1}H-NMR (300 MHz, DMSO-d_6) product **3f**.

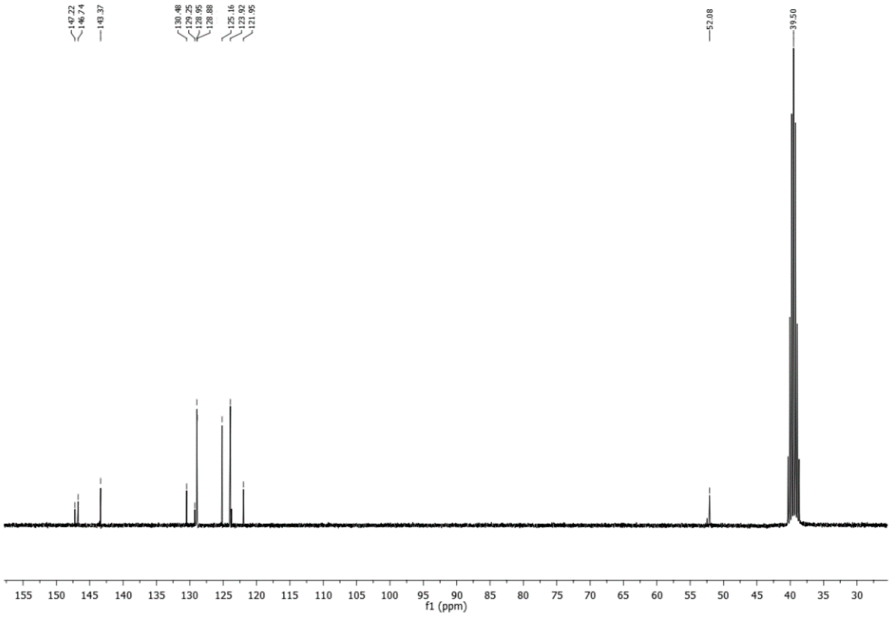

Figure S12. ^{13}C-NMR (75 MHz, DMSO-d_6) product **3f**.

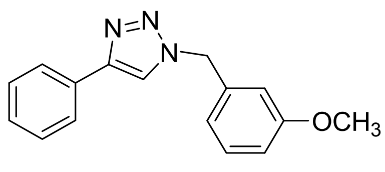

1-(3-Methoxybenzyl)-4-phenyl-1H-1,2,3-triazole (**3g**) [4]: ^{1}H-NMR (300 MHz, $CDCl_3$): δ 7.72 (2 H, d, J = 3 Hz), 7.62 (1 H, s), 7.34–7.19 (4 H, m), 6.82–6.75 (3 H, m), 5.44 (2 H, s), 3.69 (3 H, s) ppm; ^{13}C-NMR (75 MHz, $CDCl_3$): δ 159.1, 144.7, 135.1, 129.2, 127.8, 127.1, 124.7, 124.6, 123.3, 119.2, 113.2, 112.6, 54.3, 53.2 ppm. *m/z* (MALDI-TOF MS): calcd for $C_{16}H_{15}N_3O$ $[M+H]^+$: 266.1215, found: 266.1211.

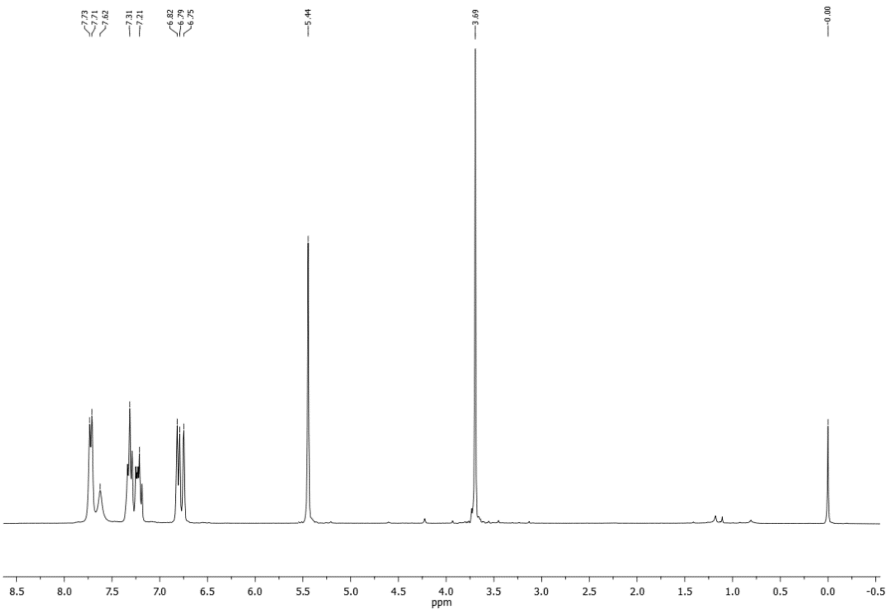

Figure S13. ^{1}H-NMR (300 MHz, $CDCl_3$) product **3g**.

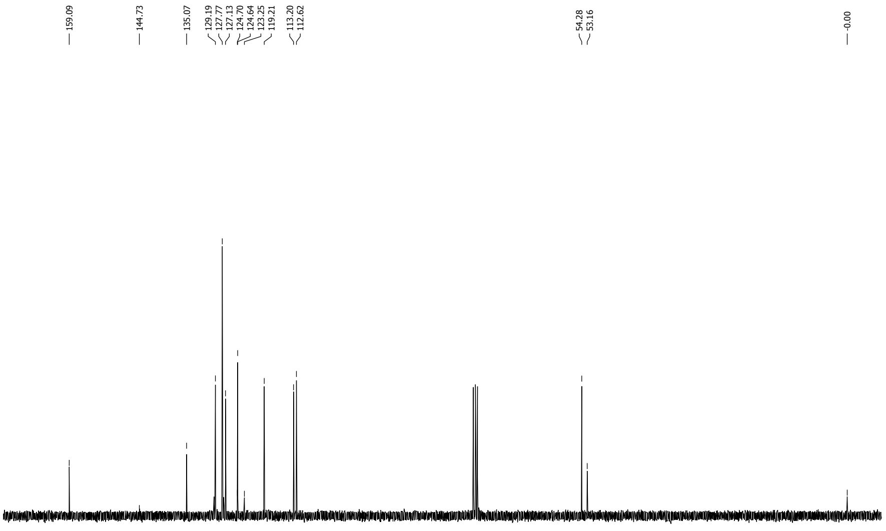

Figure S14. ^{13}C-NMR (75 MHz, $CDCl_3$) product **3g**.

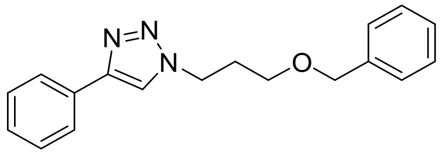

1-(3-(Benzyloxy)propyl)-4-phenyl-1H-1,2,3-triazole (**3h**): ^{1}H-NMR (300 MHz, $CDCl_3$): δ = 7.70 (2 H, d, *J* = 6 Hz), 7.55 (1 H, s), 7.35–7.21 (8 H, m), 4.44–4.41 ppm (4 H, m), 4.39 (2 H, t, *J* = 6 Hz), 2.13 (2 H, m) ppm; ^{13}C-NMR (75 MHz, $CDCl_3$): δ = 147.6, 138.0, 130.7, 128.9, 128.6, 128.5, 128.1, 127.9, 125.7, 120.2, 73.25, 66.2, 47.4, 30.5 ppm. *m/z* (MALDI-TOF MS): calcd for $C_{18}H_{19}N_3O$ $[M+H]^+$: 294.1528, found: 294.1525.

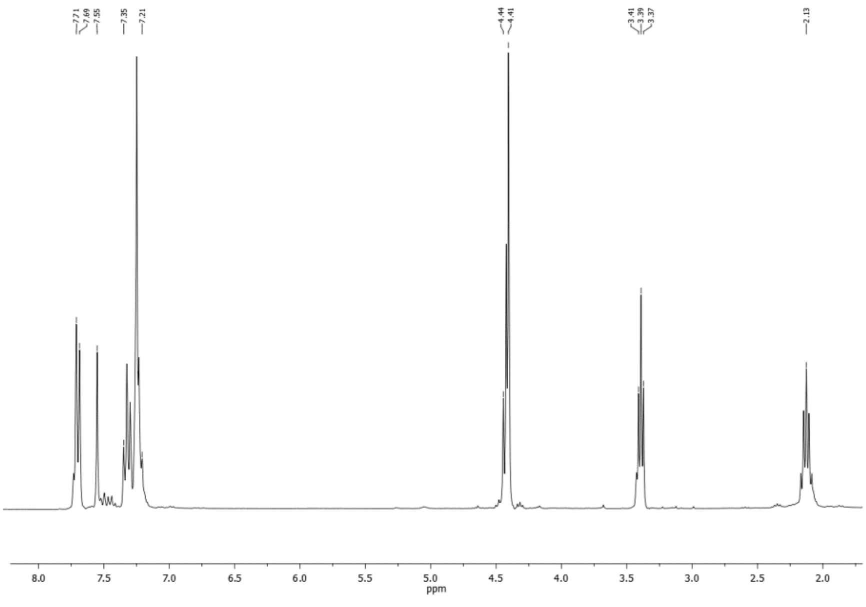

Figure S15. ^{1}H-NMR (300 MHz, $CDCl_3$) product **3h**.

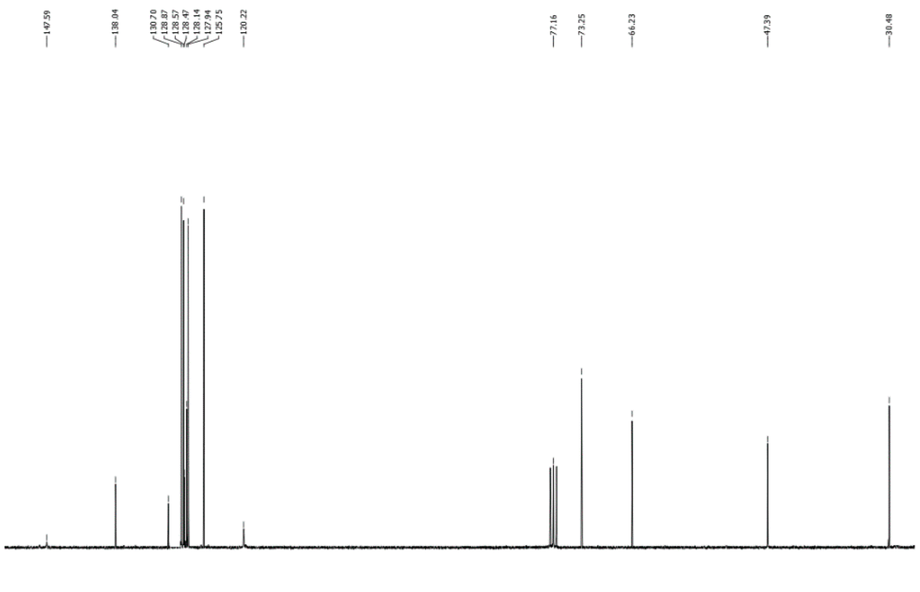

Figure S16. ^{13}C-NMR (75 MHz, $CDCl_3$) product **3h**.

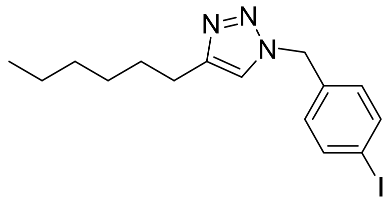

1-(4-Iodobenzyl)-4-hexyl-1H-1,2,3-triazole (**3i**): ^{1}H-NMR (300 MHz, $CDCl_3$): δ 7.68 (2 H, d, *J* = 8.2 Hz), 7.17 (1 H, s), 6.98 (2 H, d, *J* = 8.0 Hz), 5.42 (2 H, s), 2.67 (2 H, t, *J* = 7.7 Hz), 1.62 (2 H, quint, *J* = 6.1 Hz), 1.29 (6 H, m), 0.86 (3 H, t, *J* = 6.3 Hz) ppm; ^{13}C-NMR (75 MHz, $CDCl_3$): δ 149.6, 138.6, 135.1, 130.1, 120.8, 94.8, 53.8, 31.9, 29.7, 29.3, 26.1, 22.9, 14.4 ppm. *m/z* (MALDI-TOF MS): calcd for $C_{15}H_{20}IN_3$ $[M+H]^+$: 370.0701, found: 370.0702.

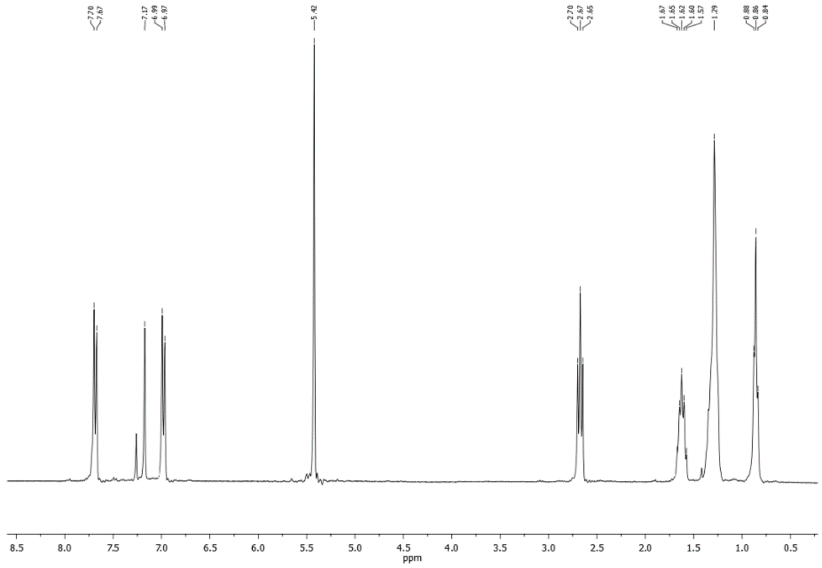

Figure S17. ^{1}H-NMR (300 MHz, $CDCl_3$) product **3i**.

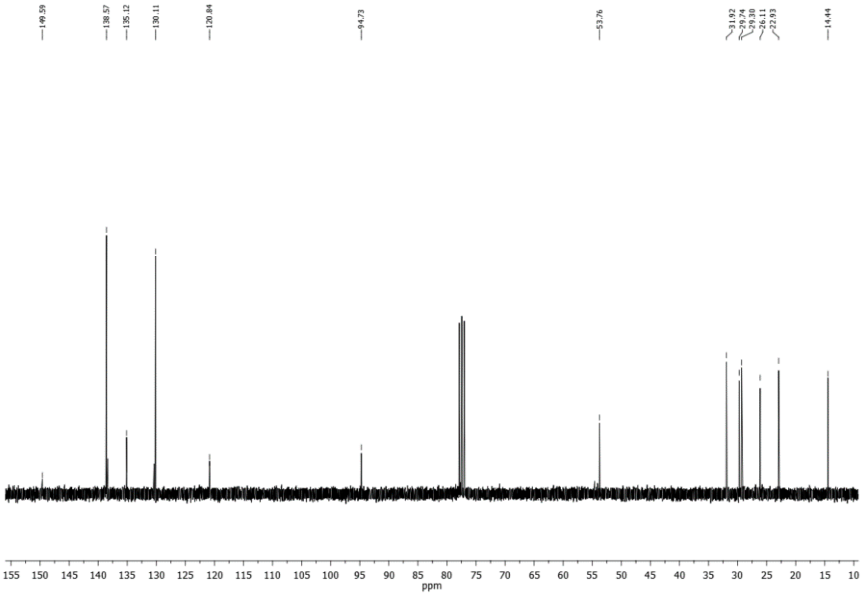

Figure S18. ^{13}C-NMR (75 MHz, $CDCl_3$) product **3i**.

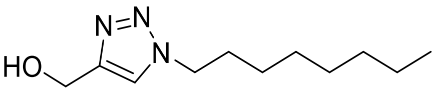

(1-Octyl-1H-1,2,3-triazol-4-yl)methanol (**3j**) [**Error! Bookmark not defined.**]: ^{1}H-NMR (300 MHz, $CDCl_3$): δ 7.48 (1 H, s), 4.71 (2 H, s), 4.26 (2 H, t, J = 9 Hz), 3.38 (1 H, b s), 1.82 (2H, m), 1.32–1.11 (10 H, m), 0.80 (3 H, t, J = 8 Hz) ppm; ^{13}C-NMR (75 MHz, $CDCl_3$): δ 148.2, 122.2, 56.5, 50.8, 32.1, 30.7, 29.4, 29.3, 26.7, 23.0, 14.4 ppm. *m/z* (MALDI-TOF MS): calcd for $C_{11}H_{21}N_3O$ [M+H]$^+$: 212.1684, found: 212.1685.

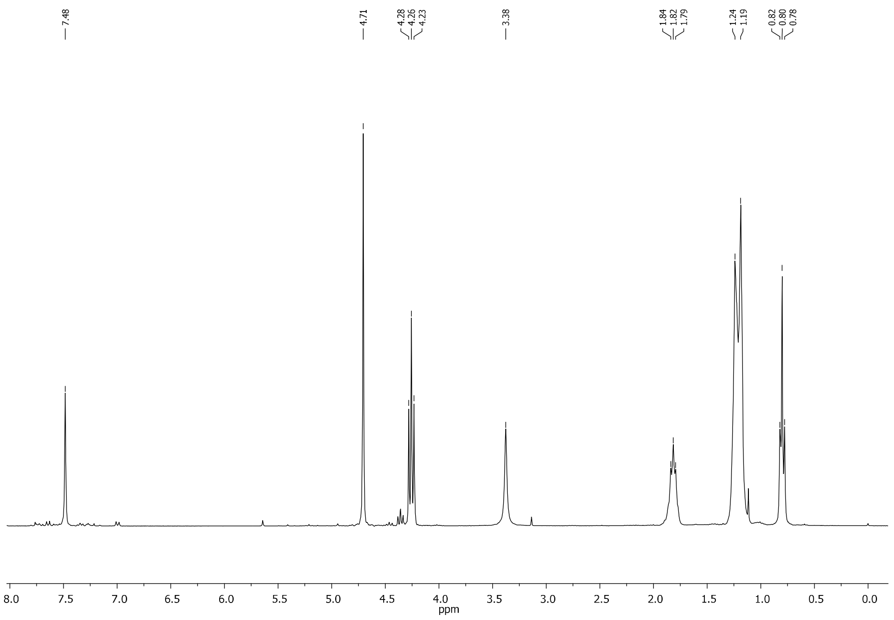

Figure S19. ^{1}H-NMR (300 MHz, $CDCl_3$) product **3j**.

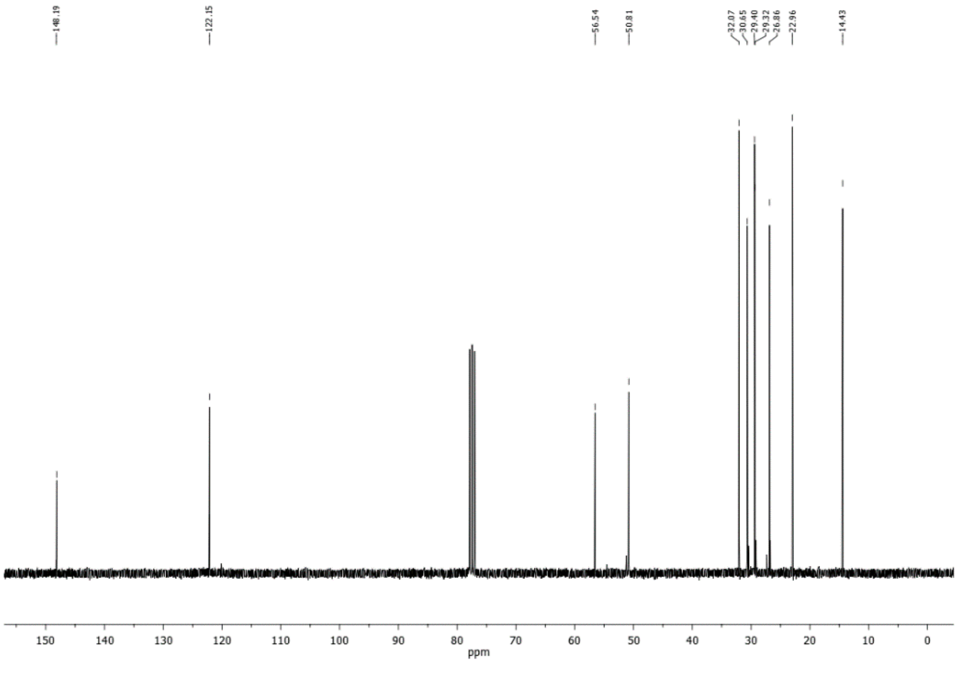

Figure S20. ^{13}C-NMR (75 MHz, $CDCl_3$) product **3j**.

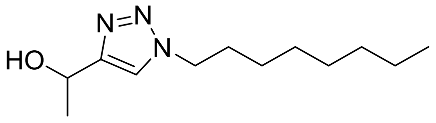

1-(1-octyl-1H-1,2,3-triazol-4-yl)ethanol (**3k**): ^{1}H-NMR (300 MHz, $CDCl_3$): δ 7.48 (1 H, s), 5.08 (1 H, b s), 4.32 (2 H, t, *J* = 9 Hz), 1.88 (2 H, m), 1.58 (3 H, d, *J* = 6 Hz), 1.35–1.14 (10 H, m), 0.87 (3 H, t, *J* = 8 Hz) ppm; ^{13}C-NMR (75 MHz, $CDCl_3$): δ 148.2, 122.2, 63.0, 50.4, 32.1, 30.6, 29.4, 29.3, 26.8, 23.1, 22.9, 14.5 ppm. *m/z* (MALDI-TOF MS): calcd for $C_{12}H_{23}N_3O$ $[M+H]^+$: 226.1841, found: 226.1839.

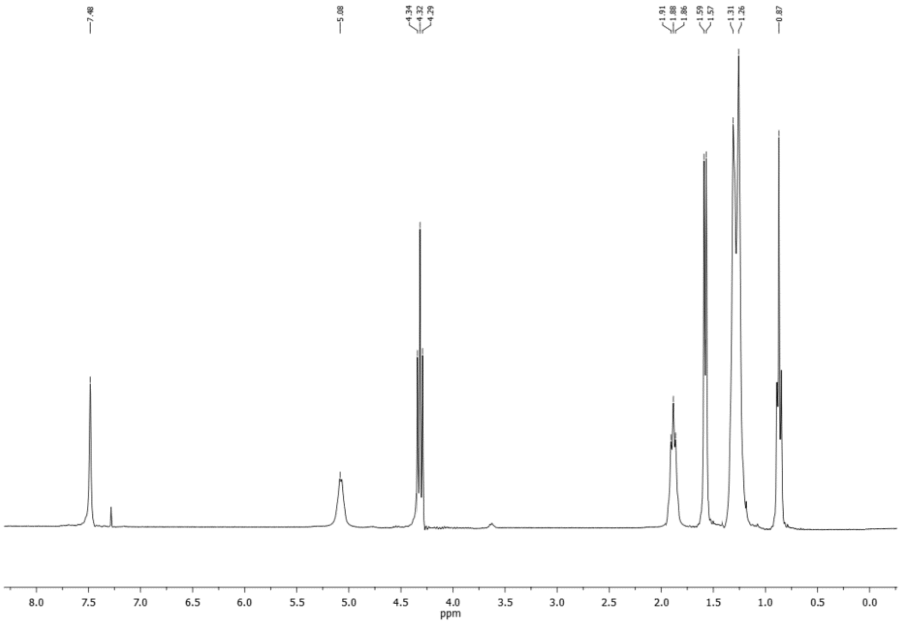

Figure S21. ^{1}H-NMR (300 MHz, $CDCl_3$) product **3k**.

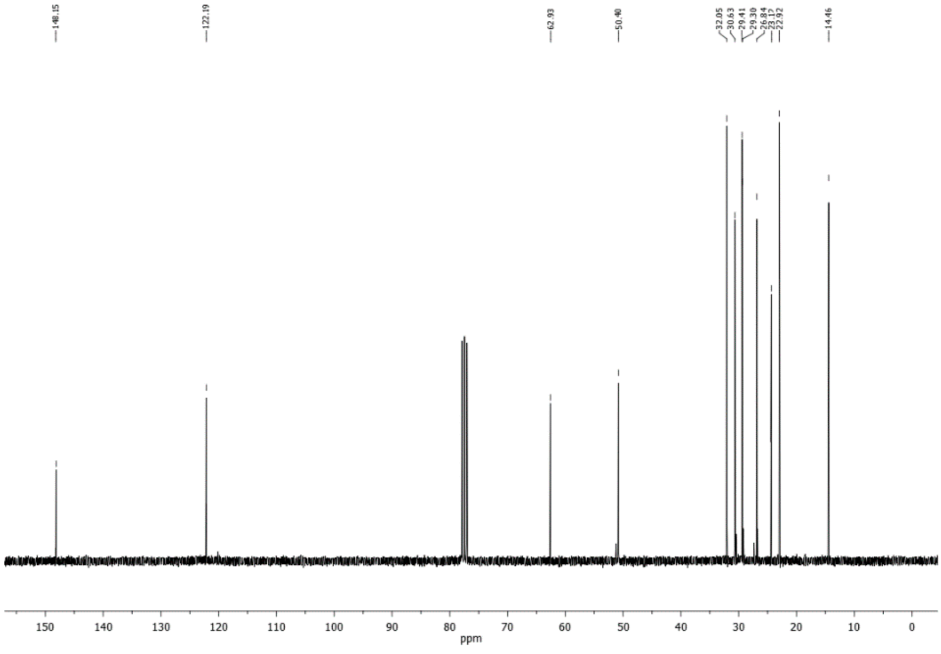

Figure S22. ^{13}C-NMR (75 MHz, $CDCl_3$) product **3k**.

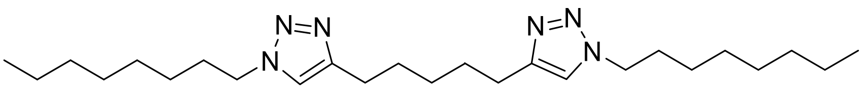

1-Octyl-4-(5-(1-octyl-1H-1,2,3-triazol-4-yl)pentyl)-1H-1,2,3-triazole (**3l**): ^{1}H-NMR (300 MHz, $CDCl_3$): δ 7.20 (2 H, s), 4.23 (4 H, t, J = 6 Hz), 2.77 (4 H, t, J = 6 Hz), 1.81 (4 H, m), 1.64 (4 H, m), 1.40–1.15 (22 H, m), 0.80 (6 H, t, J = 6 Hz) ppm; ^{13}C-NMR (75 MHz, $CDCl_3$): δ 148.3, 120.6, 50.3, 31.8, 30.4, 29.2, 29.1, 29.0, 26.6, 25.6, 22.7, 14.2 ppm. *m/z* (MALDI-TOF MS): calcd for $C_{25}H_{46}N_6$ $[M+H]^+$: 431.3783, found: 431.3782.

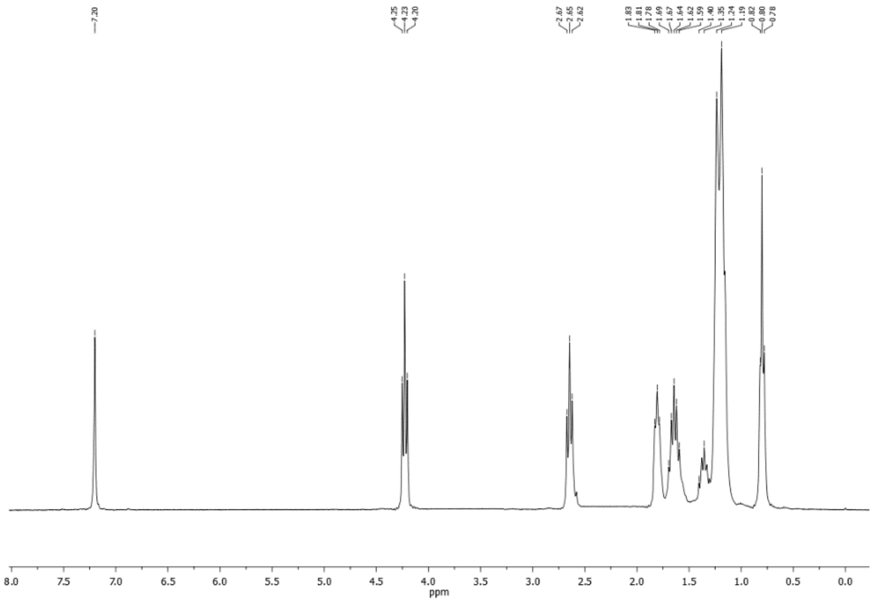

Figure S23. ^{1}H-NMR (300 MHz, $CDCl_3$) product **3l**.

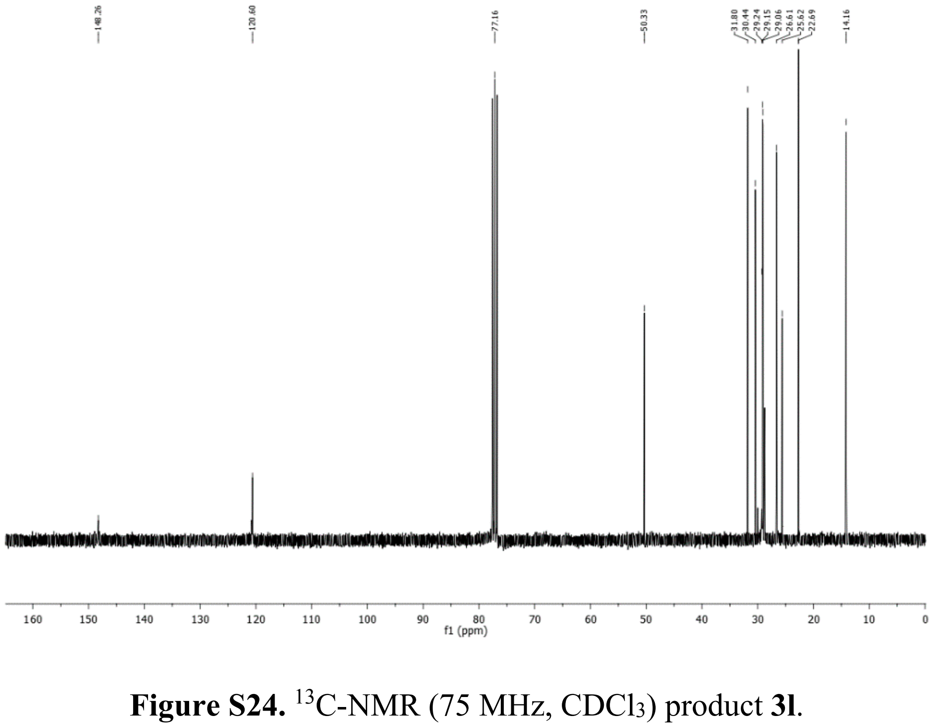

Figure S24. ^{13}C-NMR (75 MHz, $CDCl_3$) product **3l**.

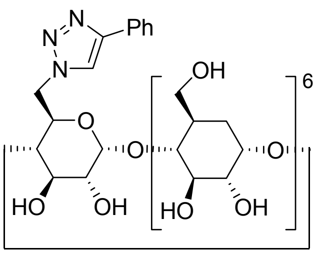

6-Monodeoxy-6-mono(4-phenyl-1H-1,2,3-triazol-1-yl)-β-CD (**3m**) [6]: ^{1}H-NMR (300 MHz, DMSO-d_6): δ 8.54 (1 H, s) 7.82 (2 H, d, J = 3 Hz), 7.43 (2 H, t, J = 9, 6.9 Hz), 7.32 (1 H, t, J = 6 Hz), 5.95–5.60 (14 H, overlapped signals), 5.08 (1 H, d, J = 3.3 Hz), 4.97 (1 H, m), 4.85 (1 H m), 4.75 (1 H, m,), 4.6–4.41 (5 H, overlapped signals), 4.25 (1 H, t, J = 6 Hz), 4.12 (1 H, t, J = 9 Hz), 3.75–3.44 (23 H, overlapped signals), 3.43–3.10 (14 H, overlapped signals), 2.91 (2 H, br). ^{13}C-NMR (DMSO-d_6, 75 MHz): δ 147.3, 131.7, 129.7, 128.6, 126.0, 122.6, 102.9, 102.0, 84.6, 83.2, 82.2, 81.4, 74–71, 70.8, 61.2, 60.7, 59.1, 51.2. ESI-MS: Calculated for $C_{50}H_{75}N_3NaO_{34}$ $[M+Na]^+$ 1284.41, found 1284.30.

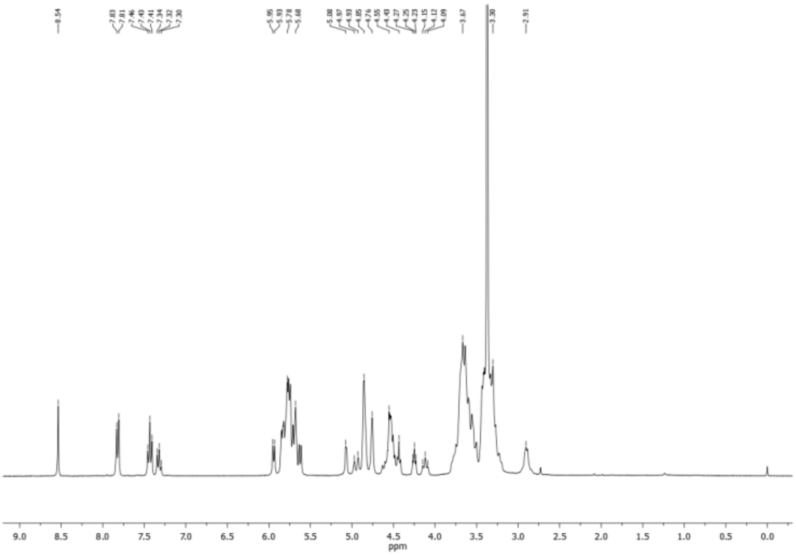

Figure S25. ^{1}H-NMR (300 MHz, DMSO-d_6) product **3m**.

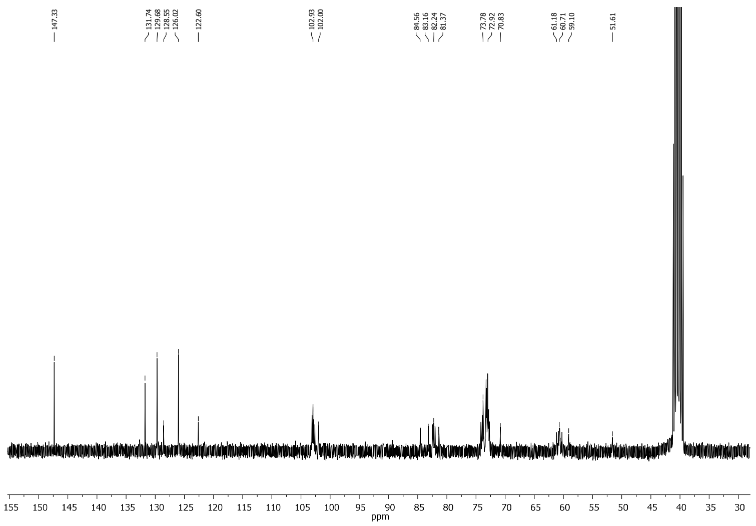

Figure S26. ^{13}C-NMR (75 MHz, DMSO-d_6) product **3m**.

References

1. Özçubukçu, S.; Ozkal, E.; Jimeno, C.; Pericàs, M.A. A Highly Active Catalyst for Huisgen 1,3-Dipolar Cycloadditions Based on the Tris(triazolyl)methanol-Cu(I) Structure. *Org. Lett.* **2009**, *11*, 4680–4683.
2. Thorwirth, R.; Stolle, A; Ondruschka, B.; Wildb, A.; Schubert, U.S. Fast, ligand- and solvent-free copper-catalyzed click reactions in a ball mill. *Chem. Commun.* **2011**, *47*, 4370–4372.
3. Gu, S.; Xua, D.; Chen, W. Heterobimetallic complexes containing an *N*-heterocyclic carbene based multidentate ligand and catalyzed tandem click/Sonogashira reactions. *Dalton Trans.* **2011**, *40*, 1576–1583.
4. Abu-Orabi, S.T.; Atfah, M.A.; Jibril, I.; Mari'i, F.M.; Ali, A.A.-S. Dipolar cycloaddition reactions of organic azides with some acetylenic compounds. *J. Heterocycl. Chem*. **1989**, *26*, 1461–1468.
5. Nasir Baiga, R.B.; Varma, R.S. Copper on chitosan: A recyclable heterogeneous catalyst for azide-alkyne cycloaddition reactions in water. *Green Chem*. **2013**, *15*, 1839–1843.
6. Cravotto, G.; Fokin, V.V.; Garella, D.; Binello, A.; Boffa, L.; Barge, A. Ultrasound-Promoted Copper-Catalyzed Azide-Alkyne Cycloaddition. *J. Comb. Chem.* **2010**, *12*, 13–15.
